# Interventions to Improve the Oral Hygiene of Individuals with Alzheimer’s Disease: A Systematic Review

**DOI:** 10.3390/dj10050092

**Published:** 2022-05-23

**Authors:** Akram Hernández-Vásquez, Antonio Barrenechea-Pulache, Rubén Aguirre-Ipenza, Daniel Comandé, Diego Azañedo

**Affiliations:** 1 Centro de Excelencia en Investigaciones Económicas y Sociales en Salud, Vicerrectorado de Investigación, Universidad San Ignacio de Loyola, Lima 15024, Peru; 2 Facultad de Ciencias de la Salud, Universidad Científica del Sur, Lima 15067, Peru; abarrenechea@cientifica.edu.pe (A.B.-P.); dazanedo@cientifica.edu.pe (D.A.); 3 Facultad de Ciencias de la Salud, Universidad Continental, Lima 15304, Peru; ruben.aguirre.ipenza@gmail.com; 4 Instituto de Efectividad Clínica y Sanitaria (IECS-CONICET), Buenos Aires C1414CPV, Argentina; dcomande@iecs.org.ar

**Keywords:** Alzheimer’s disease, dementia, oral health, oral hygiene, dental care for disabled, dental treatment

## Abstract

This systematic review evaluates published evidence on oral hygiene interventions conducted in Alzheimer’s disease (AD) patients. PubMed, Embase, Cochrane Library, CINAHL, Dentistry & Oral Sciences Source, and Web of Science were searched for articles published up to 19 April 2021. The main outcomes of interest were the Plaque index score (PI), oral health knowledge of participants or their caregivers, and behaviors and attitudes towards oral hygiene. Study quality was assessed using the Quality Assessment Tool for Observational Cohort and Cross-Sectional Studies of the National Institutes of Health. The study was conducted under PROSPERO registration code CRD42021247733. Two studies met the inclusion criteria. One was a pre-post study conducted in Brazil, and the other was a prospective cohort study carried out in China. The sample sizes of these studies were 29 and 168, respectively. Both studies were carried out in institutionalized patients and presented a significant loss to follow-up. The PI and gingival index scores both improved after the application of the respective interventions, yet the differing methodologies used precluded further comparisons. The studies were deemed to be of good and regular quality, respectively. Despite the need for more comprehensive interventions to ensure a better oral health status and a higher quality of life for AD patients, an alarming lack of studies have been conducted in this population.

## 1. Introduction

Alzheimer’s disease (AD) is the cause of 60 to 70% of cases of dementia [1]. According to the World Health Organization (WHO), in 2019, AD was the seventh leading cause of death worldwide [2]. Likewise, in 2016, an estimated 44 million people had been diagnosed with AD or other dementias, and in the same year, close to 29 million disability-adjusted life years were attributed to these diseases [3]. Due to the neurodegenerative evolution of AD, these patients may eventually experience losses in memory, complex thought processes, behaviors, and the ability to carry out daily activities [4]. Thus, ensuring comprehensive healthcare support for these patients is essential.

As the disease progresses, self-care is seriously compromised [5]. Many AD patients may find it impossible to maintain adequate oral hygiene habits, such as teeth brushing, flossing, and making periodic visits to the dentist [6]. Behavior control often becomes the focus of caregivers, and the oral hygiene of increasingly dependent patients, who are oftentimes unable to communicate oral health problems, is neglected [7]. Thus, these patients experience poorer oral hygiene outcomes with higher frequencies of coronal and root cavities [8], periodontal issues such as gingival bleeding and dental mobility, xerostomia, stomatitis, and oral candidiasis compared to people without the disease [5,9,10]. Unaddressed pain from poor oral health can lead to further deterioration of behavior. While plaque accumulation and a deteriorated periodontal status contribute to aspiration pneumonia, the hematogenous dissemination of oral bacteria can also lead to potentially fatal infections [7,11]. Likewise, periodontal diseases pose a greater risk for the development of diabetes and cardiovascular diseases [12,13].

Interventions to improve oral hygiene in AD patients are important to reduce the burden of oral diseases in this population. These interventions should be aimed at improving the adoption of usual oral hygiene measures, accompanied by strategies to increase the patient’s knowledge about their disease and its repercussions on oral health. While AD is mild, some authors suggest focusing on comprehensive oral examinations and the use of long-lasting restoration, coupled with education on a proper oral hygiene routine and dietary recommendations [5,7]. Caregivers must also be educated, as they will progressively take over the oral care routine. The introduction of electric toothbrushes, a water flosser, or a Collis Curve toothbrush can also help deliver better oral care. As the disease progresses and compliance decreases, the focus shifts to palliative care [5,7]. Thus, early interventions should positively modify patients’ and caregivers’ behaviors to improve care skills and promote the achievement of a better quality of life. 

To date, scientific publications on oral hygiene interventions among AD patients have mostly been limited to review articles or broad evaluations of populations with dementia or cognitive decline [7,14]. Thus, the objective of this study was to conduct a systematic review of the available evidence on interventions aimed at improving the oral hygiene of AD patients. Our results may aid clinical dentists and caregivers involved in the oral health care of AD patients to select the best interventions to optimize results. Likewise, gaps in knowledge and areas that require further investigation on the subject are presented.

## 2. Materials and Methods

This systematic review is reported according to the Preferred Reporting Items for Systematic Reviews and Meta-Analyses (PRISMA) statements and is registered in the PROSPERO database (registration code: CRD42021247733) [15].

### 2.1. Study Inclusion and Exclusion Criteria

Inclusion and exclusion criteria were established with the PICO method (Population, Intervention, Comparator and Outcome). Study types considered for inclusion were randomized controlled trials (RCTs), nonrandomized controlled trials, controlled before–after studies, interrupted time-series studies, and comparative observational studies. These studies involved the evaluation of methods of oral hygiene, including manual or electric tooth brushing, dental flossing, the use of mouth rinses, dental prophylaxis, oral hygiene instruction or training, and scheduled dental visits. Additionally, oral-hygiene-related knowledge-based interventions, oral-hygiene-related behavioral interventions, and oral-hygiene-related skills training for patients or caregivers were considered. Studies published in English, Spanish, or Portuguese that included participants with a diagnosis of AD from inception up to 19 April 2021 were eligible for inclusion. Review articles, systematic reviews, editorials, letters to the editor, congressional acts, and commentaries were excluded.

### 2.2. Data Sources

Six databases were searched to identify studies of interest: PubMed, Embase, Cochrane Library, CINAHL, Dentistry & Oral Sciences Source, and the Web of Science. According to the recommendations for using Google Scholar to conduct systematic reviews, we limited the number of results reviewed to the first 10 pages [16,17].

### 2.3. Search Strategies and Screening

A librarian with experience in medical research (DC) actively participated in the creation of the search strategies. All researchers reviewed and approved these strategies. Furthermore, searches of the reference list of all relevant articles were conducted. Publication status was not a restriction. The search strategy is referenced in the Supplementary Material.

To eliminate duplicate publications, search results were exported to EndNote X9 (Thompson and Reuters, Philadelphia, PA, USA). Afterwards, two of the authors (AHV and ABP) independently screened the titles and/or abstracts of the retrieved publications to identify studies that potentially met the inclusion criteria. This was achieved with the Rayyan application (https://rayyan.qcri.org/, accessed on 2 April 2022), which allows the classification of publications as included, excluded, or maybe. Conflicts about the inclusion of any publication were resolved by discussion among the reviewers. 

All studies included by titles and abstracts entered the full-text evaluation phase. These studies were evaluated independently by the same members of the review team. Conflicts between reviewers were resolved by discussion. 

### 2.4. Data Extraction

The relevant data were independently extracted by two authors (RAI and ABP) using a standardized data extraction sheet. Any disagreements were resolved through discussion with a third author (DA). The data extracted from each study included the date of publication, first author, journal name, type of publication, study objectives, period of data recollection, country, size and sample characteristics, intervention details, outcomes, and limitations. The main outcomes of interest were the Plaque index (PI) score, oral health knowledge (participants or their caregivers) and behaviors, and attitudes towards oral hygiene (participants or their caregivers). Additional outcomes of interest were gingival index scores, bacterial counts (using colony forming units), quality of life or oral health-related quality of life (i.e., Oral Health Impact Profile, or others.), caries measurements (i.e., decayed, missing, and filled teeth, the International Caries Detection and Assessment System, or other caries indices), the periodontal pocket depth (PPD), and bleeding on probing.

### 2.5. Quality Assessment

The Quality Assessment Tool for Observational Cohort and Cross-Sectional Studies of the National Institutes of Health (NIH) was used by two reviewers (AHV and ABP) to independently evaluate the quality of the studies included [18]. This instrument uses 14 criteria to evaluate the risk of bias in four principal domains: selection, information, measure, and confusion. Each evaluation criterion can be answered with one of five response categories: (“Yes” if the study complies with the criteria; “No” when the criteria are not met; and “NA”, “ND”, or “NR” when the criteria do not apply to the study, are not possible to determine, or are not reported, respectively. To determine the overall quality of the included studies, we used a previously reported methodology [19,20]. Thus, each criterion assigned “Yes” contributed one point to the overall score and criteria evaluation, while “No” and “ND” did not contribute points (0 points). Criteria qualified as “NA” were not considered for the total percentage of points. The quality of each study was determined by the percentage of the maximum score achieved (>50% good, 30–50% regular, <30% bad). 

### 2.6. Data Synthesis

A narrative synthesis focus was used. Summary tables were employed to present findings. Descriptive tables were constructed with information on oral health care interventions among patients diagnosed with AD. Descriptive summaries of the results for AD patients, including country, sex, age, intervention, and outcomes, are reported.

### 2.7. Ethical Considerations

This study did not require the approval of an ethics committee, since this was an analysis of secondary aggregate data that are publicly available and do not allow the identification of the participants studied.

## 3. Results

**Figure 1:**
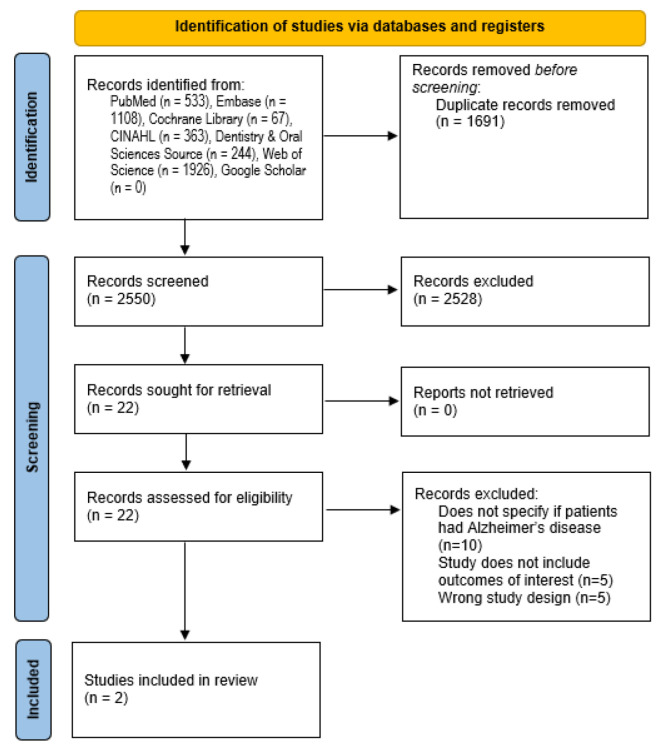
PRISMA 2020 flow diagram of the study selection process [15].

We obtained a total of 2550 results from the search after eliminating duplicates. After the title and abstract screening, 22 articles were included in the full-text review. Only two of these had been conducted specifically on AD patients and evaluated oral health interventions while measuring outcomes of interest (Figure 1).

### 3.1. Study Characteristics 

Of the studies included, one study was published in 2014 and the other in 2020. One had a pre-post study design and was conducted in Brazil [21], and the other was a prospective cohort study carried out in China [22]. No multicounty studies were included. The sample sizes of these studies were 29 and 168, respectively. The first study evaluated the effects of dental treatment and oral hygiene instructions on orofacial pain, dental characteristics, and associated factors in patients with AD over a six-month period, with measurements carried out at baseline and at one and six months. The second study evaluated the effect of a caregiver training program on the oral hygiene of formal caregivers and patients with AD over a six-week period with measurements made at baseline and every two weeks thereafter. This study included a control group that was only exposed to a 15-min oral hygiene instruction video on the role of plaque in dental disease and the modified bass brushing method, a limited training group that received an additional one-on-one training session to further perfect the aforementioned bushing method at baseline, and a comprehensive training group which received further training in weeks 2 and 4. Regarding loss to follow-up, the study by Rolim et al. lost 15 of the 29 patients initially included, while the study by Zhang lost 22 of 168 the patients over the study period. Both studies were published in English. Only the latter study specified information on the age, sex, and prior oral hygiene of the participants (Table 1).

### 3.2. Results of the Plaque Index

Both studies included the PI results. Zhang et al. identified a baseline index of 3.86 ± 0.80 in the control group, 3.84 ± 0.64 in the limited training group, and 3.77 ± 0.66 in the comprehensive training group. After six weeks of follow-up, the PI of each group had decreased to 3.20 ± 0.68, 3.03 ± 0.59, and 2.46 ± 0.52, respectively. The PI of the comprehensive training group was statistically different from the means of the other two groups and from the same group at week four, while the PIs of the limited and control group were statistically different from the means of the same groups at week four [22]. Similarly, Rolim et al. obtained measurements of 73.57 ± 5.69 (0.0–100.0) at baseline and 60.0 ± 31.62 (20.0–100.0) after 6 months of follow-up [21], and this difference was statistically significant (*p* < 0.001) (Table 2).

### 3.3. Secondary Outcomes of Interest

The gingival index (GI) was reported by Zhang et al., who found that the baseline GI was 1.92 ± 0.28, 1.97 ± 0.36, and 1.95 ± 0.31 in the control, limited training, and comprehensive training groups, respectively [22]. After six weeks of follow-up, these values fell to 1.64 ± 0.34, 1.51 ± 0.39, and 1.24 ± 0.24, respectively. After this time, the results in the comprehensive training group were statistically different from the means of the other two groups and for the same group at week four, while the GIs of the limited and control groups were statistically different from the means of the same groups at week four. Decayed, missing, filled teeth (DMFT), the oral health impact profile index (OHIP), the medium clinical attachment level (CAL), and the medium PPD were all reported by Rolim et al. The DMFT was 27.17 ± 5.69 (11–32) at baseline, and after six months of follow-up, it reached 27.50 ± 7.54 (11–32) with the difference being statistically significant (*p* < 0.001) [21]. The OHIP was 3.49 ± 6.27 (0.00–23.21) at baseline, and after six months, it reached 0.97 ± 3.49 (0.00–13.20), and the difference was statistically significant (*p* = 0.009). The medium CAL and the medium PPD were 5.88 ± 2.58 (2.0–12.0) and 1.57 ± 0.69 (0.5–3.4), respectively, during the initial evaluation and reached 2.77 ± 1.27 (1.1–4.0) and 1.55 ± 0.40 (1.0–2.0), respectively, after six months of follow-up. The differences observed in the medium PPD were statistically significant (*p* = 0.0024), but the same was not true for the medium CAL (*p* = 0.446).

### 3.4. Study Quality

The study by Zhang et al. was deemed to be of good quality (>50% of the maximum score utilizing the Quality Assessment Tool for Observational Cohort and Cross-Sectional Studies of the NIH), while the study by Rolim et al. was found to be of regular quality (50%) (Table 3).

## 4. Discussion

We intended to conduct a systematic review of studies that evaluated different interventions meant to improve oral hygiene among patients with AD. The primary outcomes of interest were the PI, oral health knowledge, behavior, and attitudes towards oral hygiene. After reviewing a total of 2550 articles, we identified only two that met the inclusion criteria. Both studies included a small sample size of institutionalized patients and presented loss to follow-up. Only one study included a comparison group. The study by Rolim et al. had the longest follow-up period of 6 months. Both studies found improvements in oral health after the intervention, but only the study by Zhang et al. was deemed to be of good quality. 

Both studies included improvements identified after the implementation of the oral health intervention. Zhang et al. identified that caregivers and patients who were part of the comprehensive learning group presented the best results in terms of the PI after 6 weeks of follow-up. Indeed, two other studies, one conducted in professional caregivers in nursing homes and one among guardians of preschool children, also found that educational interventions improve knowledge, attitudes towards oral health care, and the oral health of both guardians and their wards [23,24]. Likewise, Rolim et al. identified improvements in all outcomes evaluated after conducting repeated oral health interventions over a period of six months. This is in accordance with the established recommendations of regular visits to the dentist during the early stages of the disease to avoid requiring more complex interventions, shifting the focus towards maintenance as compliance decreases [5,7]. Despite this, it is difficult to draw conclusions from only two studies, especially given that they included small samples with notable losses to follow-up. Over half of the subjects in the study by Rolim et al. dropped out from the study due to sickness or death, while Zhang et al. recorded losses in all three study groups, with the highest number of dropouts occurring in the comprehensive group. 

The studies identified involved institutionalized patients under the care of a professional caregiver and had a maximum follow-up period of 6 months. Zhang et al. conducted a clinical trial with caregivers who had received formal training to care for elders, but this is not the norm. Most patients are cared for by informal caregivers with only empirical experience and limited access to additional resources. Around 13% of adults aged 50 years and over that live in Organization for Economic Co-operation and Development countries report the provision of informal care at least weekly [25]. Many adult caregivers suffer from their own chronic illnesses but choose to prioritize the needs of the care-recipient while facing financial strain, which limits their accessibility to resources that would improve their self-care [26]. Caregivers in low- and middle-income countries that have poorly developed long-term care systems are especially susceptible to adverse outcomes [27]. Furthermore, given that the progression of cognitive impairment affects both oral health and the use of dental services [28], the studies identified do not allow the establishment of whether the interventions are equally applicable in AD patients across the spectrum of disease. Thus, more pragmatic studies that follow patients throughout the course of their disease are required.

What is most alarming about our findings is the lack of high-quality studies that have addressed this aspect of AD patient care. There is currently a demand for further support in the care of the cognitively impaired. A systematic review of the needs of family caregivers of dementia patients concluded that support, personalized information, and education regarding care for loved ones are three of the main themes of caregiver requirements [29]. Oral health has been found to be significantly poorer in dementia patients than in healthy controls [30]. Given the detrimental effects that poor oral health has on patient wellbeing and overall survival, more research should be dedicated to devise a comprehensive strategy that ensures the best quality of life for individuals that suffer from this debilitating disease. Despite warnings by previous authors, little progress has been made to establish the best oral health care methods for cognitively impaired patients [14].

Among the limitations of our study, we must highlight the limited number of studies that were included. This is likely due to our inclusion of studies that evaluated patients diagnosed with AD and involved specific oral health outcomes. Nevertheless, AD is the most common form of dementia worldwide, and more interventional studies should aim to evaluate standardized outcomes that are relevant to clinical dentists. The few studies identified have different methodologies and are of differing levels of quality, which prevents further comparisons. Finally, we were unable to identify studies conducted in a community setting, suggesting that informal caregivers are being overlooked in this field of research. 

## 5. Conclusions

There is still an alarming lack of published studies on the use of oral health interventions among patients with AD. This prevents the development of recommendations that may prove to be useful in clinical practice. The studies included in this systematic review were conducted in institutionalized patients, had small sample sizes, and did not stratify results by the severity of the disease. In the future, longitudinal studies with standardized oral health outcomes are required to provide guidance that conforms to the needs of both patients and caregivers in specific settings. These studies should consider comprehensive and sustainable strategies based on the patients’ cognitive levels and baseline oral hygiene practices and be based in community settings.

## Figures and Tables

**Table 1:** Summary of the studies included on oral health care interventions in patients with Alzheimer’s disease.

Author (Year)	Design	Intervention	Population Description	Setting	Inclusion Criteria	Age—Mean (SD)	Sex	Prior Oral Hygiene Practices
Zhang J (2021)	Single-blinded prospective cohort study	Caregiver training	Patients with AD and caregivers in nursing homes in the Greater Zhengzhou Area, China	Nursing homes	Patient: resident for at least 3 months No treatment with antibiotics in the previous 6 weeks. No periodontal treatment in the past three months. Ramfjord teeth or neighboring substitutes were present or 1 or 2 Ramfjord teeth or their substitutes had a probing depth >4 mm. Diagnosis of AD by a neurologist from a class A tertiary care hospital. Patients deemed to be incapable of conducting effective oral hygiene Caregiver: all caregivers must have obtained a senior care certificate on senior care-related knowledge and skills	Patients: Control 76.8 (3.65) limited 75.86 (4.89) comprehensive 76.41 (3.82) Caregivers: Control 33.47 (3.95) limited: 33.86 (3.86) comprehensive 34.02 (4.27)	Patients: control: M/F ratio 15/36 limited: M/F ratio 13/36 comprehensive: M/F ratio 11/35 Caregivers: Not specified	Patients: **Regular brushing (%)** Control 9.80 Limited 8.16 Comprehensive 10.87 **No self brushing (%)** Control 56.86 Limited 57.14 Comprehensive 54.35 **Regular flossing (%)** Control 0 Limited 0 Comprehensive 0 Caregivers: **Regular brushing (%)** Control 100 Limited 100 Comprehensive 100 **No self brushing (%)** Control 0 Limited 0 Comprehensive 0 **Regular flossing (%)** Control 11.76 Limited 8.16 Comprehensive 10.87
Rolim T de S et al. (2014)	Pre-post study design	Dental treatment (all patients received oral hygiene instruction)	Patients with mild AD in Brazil	Not specified	Diagnosis of AD according to the NINCDS-ADRDA9, score between 18 and 26 in the Mini Mental State Exam (MMSE), characterizing mild AD10. The diagnosis was performed by a trained neurologist	Not specified	Not specified	Not specified

**Table 2:** Results of the studies included on oral health care interventions in patients with Alzheimer’s disease.

Author (Year)	Intervention/Duration of Intervention	Oral Hygiene Modality	Oral Health Outcomes	Time Points Measured	Intervention Key Results: mean ± SD (Range)
Zhang J (2021)	A 15-min instruction video on oral hygiene was given to all participants: It explained how dental plaque contributes to the development of periodontal disease and dental caries as well as the modified Bass method with a demonstration One-on-one training (1 h) reinforcement (5–20 min, variable between caregivers): Caregivers in the limited and comprehensive groups participated in the first training session. The hygienist demonstrated the brushing method. The caregivers brushed their own and the patients’ teeth. The hygienist analyzed the caregivers’ technical errors and made corrections; Caregivers in all groups were required to brush patients’ removable dentures daily. Only caregivers in the comprehensive group received further training. Caregivers in this group had their brushing technique examined and errors corrected during the week 2 and 4 visits. Duration of intervention: Six weeks	Modified Bass toothbrushing method: This method requires the bristles of the toothbrush to be directed toward the gingival sulcus and at a 45- degree angle to the long axis of the tooth. A horizontal back-and-forth a vibratory stroke is made with the tips of the bristles remaining in their original position, followed by a sweep toward the occlusal surface. The process should be repeated 10 times according to the study’s instructions.	Modified Quigley–Hein Plaque Index (PI) Gingival Index (GI)	Baseline, week 2, week 4, and week 6	PI Control (n = 51) Baseline: 3.86 ± 0.80 Week 2: 3.12 ± 0.76 z Week 4: 3.25 ± 0.64 z Week 6: 3.20 ± 0.68 z Limited (n = 49) Baseline: 3.84 ± 0.64 Week 2: 3.09 ± 0.71 z Week 4: 2.96 ± 0.62 *,z Week 6: 3.03 ± 0.59 z Comprehensive (n = 46) Baseline: 3.77 ± 0.66 Week 2: 3.14 ± 0.68 z Week 4: 2.76 ± 0.54 *,z Week 6: 2.46 ± 0.52 *,y, z GI Control (n = 51) Baseline: 1.92 ± 0.28 Week 2: 1.66 ± 0.32 z Week 4: 1.59 ± 0.34 z Week 6: 1.64 ± 0.34 z Limited (n = 49) Baseline: 1.97 ± 0.36 Week 2: 1.48 ± 0.38 *,z Week 4: 1.43 ± 0.38 *,z Week 6: 1.51 ± 0.39 z Comprehensive (n = 46) Baseline: 1.95 ± 0.31 Week 2: 1.53 ± 0.28 *,z Week 4: 1.32 ± 0.23 *,z Week 6: 1.24 ± 0.24 *,y, z * Statistically different from the mean of the control group at the same point. y Statistically different from the mean of the limited instruction group at the same point. z Statistically different from the mean of the previous session in the same group.
Rolim T de S et al. (2014)	Oral hygiene instructions were given to all patients, and dental treatments were given as needed: Topical nystatin Periodontal scaling and root planning Dental extraction Restoration Inferior prosthesis Periodontal surgery by modified Widman procedure Prosthesis rebasing Duration of intervention: Six months	Oral hygiene instructions Dental treatments	Plaque index (PI) DMFT, gingival bleeding index, PPD, cementoenamel junction, CAL and OHIP index	Baseline, one and six months	PI [mean ± standard deviation (range)] Initial evaluation 73.57 ± 5.69 (0.0–100.0) After one month 26.21 ± 11.64 * (8.0–50.4) After six months 60.0 ± 31.62 (20.0–100.0) *p*-value: <0.001 DMFT [mean± standard deviation (range)] Initial evaluation 27.17 ± 5.69 (11–32) After one month 23.44 ± 8.86 * (8.0–50.4) After six months 27.50 ± 7.54 (11–32) *p*-value: <0.001 OHIP index [mean± standard deviation (range)] Initial evaluation 3.49 ± 6.27 (0.00–23.21) After one month 1.87 ± 4.92 (0.00–20.09) After six months 0.97 ± 3.49 (0.00–13.20) *p*-value: 0.009 Medium CAL (mm) [mean± standard deviation (range)] Initial evaluation 2.94 ± 1.26 (1.2–5.9) After one month 2.45 ± 1.00 (1.00–4.0) After six months 2.77 ± 1.27 (1.1–4.0) *p*-value: 0.449 Medium PPD (mm) [mean ± standard deviation (range)] Initial evaluation 1.57 ± 0.69 (0.5–3.4) After one month 2.52 ± 3.34 * (1.0–12.0) After six months 1.55 ± 0.40 (1.0–2.0) *p*-value: 0.024 * Analysis of repetitive measures—statistically different from the initial and last evaluations

**Table 3:** Evaluation of the quality of the studies included (n = 2).

Author (Year)	P1	P2	P3	P4	P5	P6	P7	P8	P9	P10	P11	P12	P13	P14	Rating
Zhang J (2021)	Yes	Yes	ND	Yes	No	Yes	Yes	Yes	Yes	Yes	Yes	No	Yes	Yes	78.6%
Rolim T de S et al. (2014)	Yes	Yes	ND	Yes	No	Yes	Yes	No	Yes	Yes	No	No	No	No	50.0%

Quality Assessment Tool for Observational Cohort and Cross-Sectional Studies of the National Institute of Health.

## Data Availability

Data sharing is not applicable to this article as no new data were created or analyzed in this study. This is a systematic review of the scientific literature.

## Supplementary Materials

The following are available online at https://www.mdpi.com/article/10.3390/dj10050092/s1, Table S1: Search Strategies.
