# Supplementary material for: Interventions to Improve the Oral Hygiene of Individuals with Alzheimer’s Disease: A Systematic Review
Source: Dent J (Basel). 2022 May 23;10(5):92. doi: 10.3390/dj10050092 (PMC9139279; doi:10.3390/dj10050092)
Supplement: Supplementary file 1 [file dentistry-10-00092-s001.zip › dentistry-1692654-supplementary.pdf]

Supplementary material

Supplementary Table S1. Search Strategies

Database	PubMed<br>Date: 19/04/2021		Results
Search Strategy	#1	Oral Health[Mesh]	17,462
	#2	Oral Health[tiab]	28,498
	#3	Oral Hygiene[Mesh]	19,824
	#4	Oral Hygiene[tiab]	13,909
	#5	Tooth Brush*[tiab]	2,128
	#6	Toothbrush*[tiab]	5,873
	#7	Dental Brush*[tiab]	48
	#8	Dental Devices, Home Care[Mesh]	2,025
	#9	Dental Floss*[tiab]	713
	#10	Dental Device*[tiab]	240
	#11	Dental Cleaning[tiab]	180
	#12	Dental Hygien*[tiab]	5,26
	#13	Mouthwashes[Mesh]	7,033
	#14	Mouth Wash*[tiab]	328
	#15	Mouthwash*[tiab]	2,899
	#16	Oral Wash*[tiab]	119
	#17	Oral Rinse*[tiab]	817
	#18	Mouth Rinse*[tiab]	1,051
	#19	Dentifrices[Mesh]	6,97
	#20	Dentifrice*[tiab]	3,312
	#21	Toothpaste*[tiab]	4,573
	#22	Tooth Paste*[tiab]	229
	#23	Denture Clean*[tiab]	460
	#24	Denture Hygiene[tiab]	234
	#25	#1 OR #2 OR #3 OR #4 OR #5 OR #6 OR #7 OR #8 OR #9 OR #10 OR #11 OR #12 OR #13 OR #14 OR #15 OR #16 OR #17 OR #18 OR #19 OR #20 OR #21 OR #22 OR #23 OR #24	72,175
	#26	Dementia[Majr]	143,205
	#27	Dementia[tiab]	117,61
	#28	Amentia[tiab]	79
	#29	Alzheimer[tiab]	157,396
	#30	Cognitive Dysfunction[Mesh]	21,39
	#31	Cognitive Dysfunction*[tiab]	16,643
	#32	Cognitive Impair*[tiab]	71,432
	#33	Cognitive Disorder*[tiab]	5,051
	#34	Cognitive Decline*[tiab]	25,012
	#35	Cognitive Deterior*[tiab]	1,928
	#36	Neurocognitive Impair*[tiab]	2,506
	#37	Neurocognitive Disorder*[tiab]	2,947
	#38	Neurocognitive Decline*[tiab]	413
	#39	Neurocognitive Deterior*[tiab]	51
	#40	Mental Deterior*[tiab]	1,075

	#41	#26 OR #27 OR #28 OR #29 OR #30 OR #31 OR #32 OR #33 OR #34 OR #35 OR #36 OR #37 OR #38 OR #39 OR #40	324,1
	#42	#25 AND #41	533

Database	EMBase (Elsevier)<br>Date: 19/04/2021		Results
Search Strategy	#1	(oral NEAR/1 health):ti,ab	29,05
	#2	mouth hygiene'/exp	27,774
	#3	(oral NEAR/1 hygiene):ti,ab	14,916
	#4	(tooth NEAR/1 brush*):ti,ab	2,327
	#5	toothbrush*:ti,ab	5,691
	#6	(dental NEAR/1 brush*):ti,ab	97
	#7	dental device'/exp	146,512
	#8	(dental NEAR/1 floss*):ti,ab	802
	#9	(dental NEAR/1 device*):ti,ab	265
	#10	(dental NEAR/1 cleaning):ti,ab	219
	#11	(dental NEAR/1 hygien*):ti,ab	5,511
	#12	mouthwash'/exp	4,986
	#13	(mouth NEAR/1 wash*):ti,ab	497
	#14	mouthwash*:ti,ab	3,485
	#15	(oral NEAR/1 wash*):ti,ab	211
	#16	(oral NEAR/1 rinse*):ti,ab	977
	#17	(mouth NEAR/1 rinse*):ti,ab	1,198
	#18	toothpaste'/exp	8,691
	#19	dentifrice*:ti,ab	3,121
	#20	toothpaste*:ti,ab	4,991
	#21	(tooth NEAR/1 paste*):ti,ab	322
	#22	(denture NEAR/1 clean*):ti,ab	445
	#23	(denture NEAR/3 hygiene):ti,ab	278
	#24	#1 OR #2 OR #3 OR #4 OR #5 OR #6 OR #7 OR #8 OR #9 OR #10 OR #11 OR #12 OR #13 OR #14 OR #15 OR #16 OR #17 OR #18 OR #19 OR #20 OR #21 OR #22 OR #23	211,795
	#25	dementia'/exp/mj	230,269
	#26	dementia:ti,ab	165,979
	#27	amentia:ti,ab	93
	#28	alzheimer disease'/exp	210,189
	#29	alzheimer:ti,ab	208,778
	#30	cognitive defect'/exp	522,616
	#31	(cognitive NEAR/1 dysfunction*):ti,ab	24,492
	#32	(cognitive NEAR/1 impair*):ti,ab	112,865
	#33	(cognitive NEAR/1 disorder*):ti,ab	8,386
	#34	(cognitive NEAR/1 decline*):ti,ab	38,283
	#35	(cognitive NEAR/1 deterior*):ti,ab	3,28
	#36	(neurocognitive NEAR/1 impair*):ti,ab	4,16
	#37	(neurocognitive NEAR/1 disorder*):ti,ab	4,475
	#38	(neurocognitive NEAR/1 decline*):ti,ab	739
	#39	(neurocognitive NEAR/1 deterior*):ti,ab	94
	#40	(mental NEAR/1 deterior*):ti,ab	1,688
	#41	#25 OR #26 OR #27 OR #28 OR #29 OR #30 OR #31 OR #32 OR #33 OR #34 OR #35 OR #36 OR #37 OR #38 OR #39 OR #40	598,179
	#42	#24 AND #41	1,108

Database	Cochrane Library<br>Date: 19/04/2021		Results
Search Strategy	#1	MeSH descriptor: [Oral Health] explode all trees	429
	#2	(Oral NEAR/1 Health):ti,ab,kw	3428
	#3	MeSH descriptor: [Oral Hygiene] explode all trees	2219
	#4	(Oral NEAR/1 Hygiene):ti,ab,kw	4419
	#5	(Tooth NEAR/1 Brush*):ti,ab,kw	1445
	#6	Toothbrush*:ti,ab,kw	2955
	#7	(Dental NEAR/1 Brush*):ti,ab,kw	69
	#8	MeSH descriptor: [Dental Devices, Home Care] explode all trees	375
	#9	(Dental NEAR/1 Floss*):ti,ab,kw	506
	#10	(Dental NEAR/1 Device*):ti,ab,kw	484
	#11	(Dental NEAR/1 Cleaning):ti,ab,kw	56
	#12	(Dental NEAR/1 Hygien*):ti,ab,kw	452
	#13	MeSH descriptor: [Mouthwashes] explode all trees	1665
	#14	(Mouth NEAR/1 Wash*):ti,ab,kw	283
	#15	Mouthwash*:ti,ab,kw	3083
	#16	(Oral NEAR/1 Wash*):ti,ab,kw	38
	#17	(Oral NEAR/1 Rinse*):ti,ab,kw	369
	#18	(Mouth NEAR/1 Rinse*):ti,ab,kw	774
	#19	MeSH descriptor: [Dentifrices] explode all trees	1593
	#20	Dentifrice*:ti,ab,kw	2182
	#21	Toothpaste*:ti,ab,kw	2250
	#22	(Tooth NEAR/1 Paste*):ti,ab,kw	135
	#23	(Denture NEAR/1 Clean*):ti,ab,kw	109
	#24	(Denture NEAR/1 Hygiene):ti,ab,kw	47
	#25	#1 OR #2 OR #3 OR #4 OR #5 OR #6 OR #7 OR #8 OR #9 OR #10 OR #11 OR #12 OR #13 OR #14 OR #15 OR #16 OR #17 OR #18 OR #19 OR #20 OR #21 OR #22 OR #23 OR #24	14392
	#26	MeSH descriptor: [Dementia] explode all trees	6074
	#27	Dementia:ti,ab,kw	13261
	#28	Amentia:ti,ab,kw	1
	#29	Alzheimer:ti,ab,kw	11366
	#30	MeSH descriptor: [Cognitive Dysfunction] explode all trees	1675
	#31	(Cognitive NEAR/1 Dysfunction*):ti,ab,kw	3570
	#32	(Cognitive NEAR/1 Impair*):ti,ab,kw	9647
	#33	(Cognitive NEAR/1 Disorder*):ti,ab,kw	852
	#34	(Cognitive NEAR/1 Decline*):ti,ab,kw	2974
	#35	(Cognitive NEAR/1 Deterior*):ti,ab,kw	328
	#36	(Neurocognitive NEAR/1 Impair*):ti,ab,kw	267
	#37	(Neurocognitive NEAR/1 Disorder*):ti,ab,kw	383
	#38	(Neurocognitive NEAR/1 Declin*):ti,ab,kw	70
	#39	(Neurocognitive NEAR/1 Deterior*):ti,ab,kw	15
	#40	(Mental NEAR/1 Deterior*):ti,ab,kw	215
	#41	#26 OR #27 OR #28 OR #29 OR #30 OR #31 OR #32 OR #33 OR #34 OR #35 OR #36 OR #37 OR #38 OR #39 OR #40	30205
	#42	#25 AND #41	67

Database	CINAHL (EBSCO)<br>Date: 18/04/2021		Results
Search Strategy	#1	(MH "Oral Health")	12,932
	#2	TI (Oral N1 Health) OR AB (Oral N1 Health)	14,903
	#3	(MH "Oral Hygiene+")	7,928
	#4	TI (Oral N1 Hygiene) OR AB (Oral N1 Hygiene)	4,057
	#5	TI (Tooth N1 Brush*) OR AB (Tooth N1 Brush*)	1,265
	#6	TI Toothbrush* OR AB Toothbrush*	1,878
	#7	TI (Dental N1 Brush*) OR AB (Dental N1 Brush*)	101
	#8	(MH "Dental Devices, Home Care+")	1,58
	#9	TI (Dental N1 Floss*) OR AB (Dental N1 Floss*)	318
	#10	TI (Dental N1 Device*) OR AB (Dental N1 Device*)	140
	#11	TI (Dental N1 Cleaning) OR AB (Dental N1 Cleaning)	103
	#12	TI (Dental N1 Hygien*) OR AB (Dental N1 Hygien*)	6,266
	#13	(MH "Mouthwashes+")	2,492
	#14	TI (Mouth N1 Wash*) OR AB (Mouth N1 Wash*)	71
	#15	TI Mouthwash* OR AB Mouthwash*	882
	#16	TI (Oral N1 Wash*) OR AB (Oral N1 Wash*)	42
	#17	TI (Oral N1 Rinse*) OR AB (Oral N1 Rinse*)	328
	#18	TI (Mouth N1 Rinse*) OR AB (Mouth N1 Rinse*)	483
	#19	(MH "Dentifrices")	2,265
	#20	TI Dentifrice* OR AB Dentifrice*	663
	#21	TI Toothpaste* OR AB Toothpaste*	1,44
	#22	TI (Tooth N1 Paste*) OR AB (Tooth N1 Paste*)	42
	#23	TI (Denture N1 Clean*) OR AB (Denture N1 Clean*)	134
	#24	TI (Denture N1 Hygiene) OR AB (Denture N1 Hygiene)	77
	#25	S1 OR S2 OR S3 OR S4 OR S5 OR S6 OR S7 OR S8 OR S9 OR S10 OR S11 OR S12 OR S13 OR S14 OR S15 OR S16 OR S17 OR S18 OR S19 OR S20 OR S21 OR S22 OR S23 OR S24	23,741
	#26	(MH "Dementia+")	76,094
	#27	TI Dementia OR AB Dementia	54,557
	#28	TI Amentia OR AB Amentia	3
	#29	(MH "Alzheimer's Disease")	33,447
	#30	TI Alzheimer OR AB Alzheimer	27,55
	#31	(MH "Cognition Disorders+")	33,027
	#32	TI (Cognitive N1 Dysfunction*) OR AB (Cognitive N1 Dysfunction*)	3,946
	#33	TI (Cognitive N1 Impair*) OR AB (Cognitive N1 Impair*)	26,672
	#34	TI (Cognitive N1 Disorder*) OR AB (Cognitive N1 Disorder*)	1,791
	#35	TI (Cognitive N1 Decline*) OR AB (Cognitive N1 Decline*)	9,629
	#36	TI (Cognitive N1 Deterior*) OR AB (Cognitive N1 Deterior*)	771
	#37	TI (Neurocognitive N1 Impair*) OR AB (Neurocognitive N1 Impair*)	818
	#38	TI (Neurocognitive N1 Disorder*) OR AB (Neurocognitive N1 Disorder*)	766
	#39	TI (Neurocognitive N1 Decline*) OR AB (Neurocognitive N1 Decline*)	144
	#40	TI (Neurocognitive N1 Deterior*) OR AB (Neurocognitive N1 Deterior*)	20
	#41	TI (Mental N1 Deterior*) OR AB (Mental N1 Deterior*)	311
	#42	S26 OR S27 OR S28 OR S29 OR S30 OR S31 OR S32 OR S33 OR S34 OR S35 OR S36 OR S37 OR S38 OR S39 OR S40 OR S41	91,252
	#43	S25 AND S42	363

Database	Dentistry & Oral Sciences Source (EBSCO)<br>Date: 19/04/2021		Results
Search Strategy	#1	(MH "Oral Health")	
	#2	TI (Oral N1 Health) OR AB (Oral N1 Health)	(20,494)
	#3	(MH "Oral Hygiene+")	(0)
	#4	TI (Oral N1 Hygiene) OR AB (Oral N1 Hygiene)	(7,847)
	#5	TI (Tooth N1 Brush*) OR AB (Tooth N1 Brush*)	(2,008)
	#6	TI Toothbrush* OR AB Toothbrush*	(3,563)
	#7	TI (Dental N1 Brush*) OR AB (Dental N1 Brush*)	(168)
	#8	(MH "Dental Devices, Home Care+")	(0)
	#9	TI (Dental N1 Floss*) OR AB (Dental N1 Floss*)	(539)
	#10	TI (Dental N1 Device*) OR AB (Dental N1 Device*)	(277)
	#11	TI (Dental N1 Cleaning) OR AB (Dental N1 Cleaning)	(170)
	#12	TI (Dental N1 Hygien*) OR AB (Dental N1 Hygien*)	(9,106)
	#13	(MH "Mouthwashes+")	(0)
	#14	TI (Mouth N1 Wash*) OR AB (Mouth N1 Wash*)	(105)
	#15	TI Mouthwash* OR AB Mouthwash*	(1,303)
	#16	TI (Oral N1 Wash*) OR AB (Oral N1 Wash*)	(20)
	#17	TI (Oral N1 Rinse*) OR AB (Oral N1 Rinse*)	(340)
	#18	TI (Mouth N1 Rinse*) OR AB (Mouth N1 Rinse*)	(649)
	#19	(MH "Dentifrices")	(1,691)
	#20	TI Dentifrice* OR AB Dentifrice*	(1,584)
	#21	TI Toothpaste* OR AB Toothpaste*	(2,857)
	#22	TI (Tooth N1 Paste*) OR AB (Tooth N1 Paste*)	(152)
	#23	TI (Denture N1 Clean*) OR AB (Denture N1 Clean*)	(323)
	#24	TI (Denture N1 Hygiene) OR AB (Denture N1 Hygiene)	(164)
	#25	S1 OR S2 OR S3 OR S4 OR S5 OR S6 OR S7 OR S8 OR S9 OR S10 OR S11 OR S12 OR S13 OR S14 OR S15 OR S16 OR S17 OR S18 OR S19 OR S20 OR S21 OR S22 OR S23 OR S24	(42,150)
	#26	(MH "Dementia+")	(0)
	#27	TI Dementia OR AB Dementia	(401)
	#28	TI Amentia OR AB Amentia	(1)
	#29	(MH "Alzheimer's Disease")	(194)
	#30	TI Alzheimer OR AB Alzheimer	(273)
	#31	(MH "Cognition Disorders+")	(0)
	#32	TI (Cognitive N1 Dysfunction*) OR AB (Cognitive N1 Dysfunction*)	(56)
	#33	TI (Cognitive N1 Impair*) OR AB (Cognitive N1 Impair*)	(292)
	#34	TI (Cognitive N1 Disorder*) OR AB (Cognitive N1 Disorder*)	(0)
	#35	TI (Cognitive N1 Decline*) OR AB (Cognitive N1 Decline*)	(80)
	#36	TI (Cognitive N1 Deterior*) OR AB (Cognitive N1 Deterior*)	(12)
	#37	TI (Neurocognitive N1 Impair*) OR AB (Neurocognitive N1 Impair*)	(7)
	#38	TI (Neurocognitive N1 Disorder*) OR AB (Neurocognitive N1 Disorder*)	(6)
	#39	TI (Neurocognitive N1 Decline*) OR AB (Neurocognitive N1 Decline*)	(3)
	#40	TI (Neurocognitive N1 Deterior*) OR AB (Neurocognitive N1 Deterior*)	(0)
	#41	TI (Mental N1 Deterior*) OR AB (Mental N1 Deterior*)	(5)
	#42	S26 OR S27 OR S28 OR S29 OR S30 OR S31 OR S32 OR S33 OR S34 OR S35 OR S36 OR S37 OR S38 OR S39 OR S40 OR S41	(925)
	#43	S25 AND S42	(244)

Database	Web of Science<br>Date: 19/04/2021		Results
Search Strategy	#1	TS=Oral Health	202.433
	#2	TI=Oral Health	19.849
	#3	TS=Oral Hygiene	49.973
	#4	TI=Oral Hygiene	3.891
	#5	TI=Tooth Brush*	646
	#6	TI=Toothbrush*	3.251
	#7	TI=Dental Brush*	193
	#8	TS=Dental Devices	29.263
	#9	TI=Dental Floss*	268
	#10	Ti=Dental Device*	752
	#11	TI=Dental Cleaning	190
	#12	TI=Dental Hygien*	5.22
	#13	TS=Mouthwashes	8.121
	#14	TI=Mouth Wash*	126
	#15	TI=Mouthwash*	1.617
	#16	TI=Oral Wash*	131
	#17	TI=Oral Rinse*	451
	#18	TI=Mouth Rinse*	532
	#19	TS=Dentifrices	6.515
	#20	TI=Dentifrice*	2.63
	#21	TI=Toothpaste*	2.4
	#22	TI=Tooth Paste*	269
	#23	TI=Denture Clean*	412
	#24	TI=Denture Hygiene	102
	#25	#1 OR #2 OR #3 OR #4 OR #5 OR #6 OR #7 OR #8 OR #9 OR #10 OR #11 OR #12 OR #13 OR #14 OR #15 OR #16 OR #17 OR #18 OR #19 OR #20 OR #21 OR #22 OR #23 OR #24	266.138
	#26	TS=Dementia	224.973
	#27	TI=Dementia	78.156
	#28	TI=Amentia	69
	#29	TS=Alzheimer Disease	225.907
	#30	TI=Alzheimer	101.257
	#31	TS=Cognitive Dysfunction	78.091
	#32	TI=Cognitive Dysfunction*	6.141
	#33	TI=Cognitive Impair*	32.345
	#34	TI=Cognitive Disorder*	11.656
	#35	TI=Cognitive Decline*	8.097
	#36	TI=Cognitive Deterior*	757
	#37	TI=Neurocognitive Impair*	1.019
	#38	TI=Neurocognitive Disorder*	2.129
	#39	TI=Neurocognitive Decline*	141
	#40	TI=Neurocognitive Deterior*	17
	#41	TI=Mental Deterior*	306
	#42	#26 OR #27 OR #28 OR #29 OR #30 OR #31 OR #32 OR #33 OR #34 OR #35 OR #36 OR #37 OR #38 OR #39 OR #40 OR #41	445.241
	#43	#25 AND #42	1.926
